# A case report of radiopharmaceutical needlestick injury with scintigraphic imaging and dose quantification

**DOI:** 10.1016/j.radcr.2022.02.055

**Published:** 2022-03-24

**Authors:** James Elliott, Mariq Weatherley

**Affiliations:** a School of Allied and Public Health Professions, Canterbury Christ Church University, North Holmes Road Campus, Canterbury**,** Kent, CT1 1QU, UK; b Maidstone Nuclear Medicine Department, Maidstone and Tunbridge Wells NHS Trust, Kent, UK

**Keywords:** Preventable injury, Sharps, Radiopharmacy, Contamination, Effective dose

## Abstract

Attention to the implications of common needle stick injuries has focused heavily on the risk of cross-infection from blood-borne pathogens. An additional risk to the nuclear medicine healthcare worker is that of subcutaneous radioactive contamination from radiopharmaceuticals. This case report provides a rare opportunity to examine the clinical and operator causes of one such event during the dispensing of ^99m^Tc-Tetrofosmin. Contamination monitoring, scintigraphic imaging, and quantification of effective radiation dose provide the level of risk to the operator from the subcutaneous radioactive contamination. Findings demonstrated a very low dose to operator and no deterministic radiobiological effects. Delayed imaging demonstrated negligible biological clearance from the injury site. Implications of the findings for clinical practice are discussed, highlighting the need for a careful and calm approach to radiopharmacy activities.

## Introduction

Needlestick injuries (NSI) present healthcare workers with the risk of cross-infection from blood-borne pathogens such as hepatitis B virus, hepatitis C virus, and human immunodeficiency virus [1]. Occurrences of NSI are regrettably common both internationally and within interventional radiology departments [2,3]. However, published examples within nuclear medicine illustrating the additional risk of radionuclides are lacking. NSI events commonly occur at the point of patient interaction, thereby increasing the risk of blood-borne pathogen transmission. This case report illustrates the dangers of radiopharmaceutical NSI occurring before patient contact, during the dispensing of radionuclides from a multidose vial. The risk of radiopharmaceutical NSI is greatest to the hand and fingers during needle insertion/removal from the vial and during re-capping of needles for the safe preparation of radiopharmaceuticals. Current guidance states that re-capping prevents radioactive contamination of equipment and reduces the risks of dose to the operator [4] but does not address the associated risk of NSI. As a result, nuclear medicine staff remain remains exposed to the dangers of subcutaneous radioactive contamination typically not seen in other healthcare environments.

Previous studies have acknowledged the occurrence and risks of radiopharmaceutical NSI, highlighting the utility of re-capping devices to reduce such events [5,6]. Surprisingly, there is an absence of documented cases within the academic literature. This article aims to address this gap by outlining one case of radiopharmaceutical NSI. An exploration of causes, operator dose, and biological clearance shall be undertaken in conjunction with scintigraphic imaging and subsequent quantification.

## Case report

**Fig. 1:**
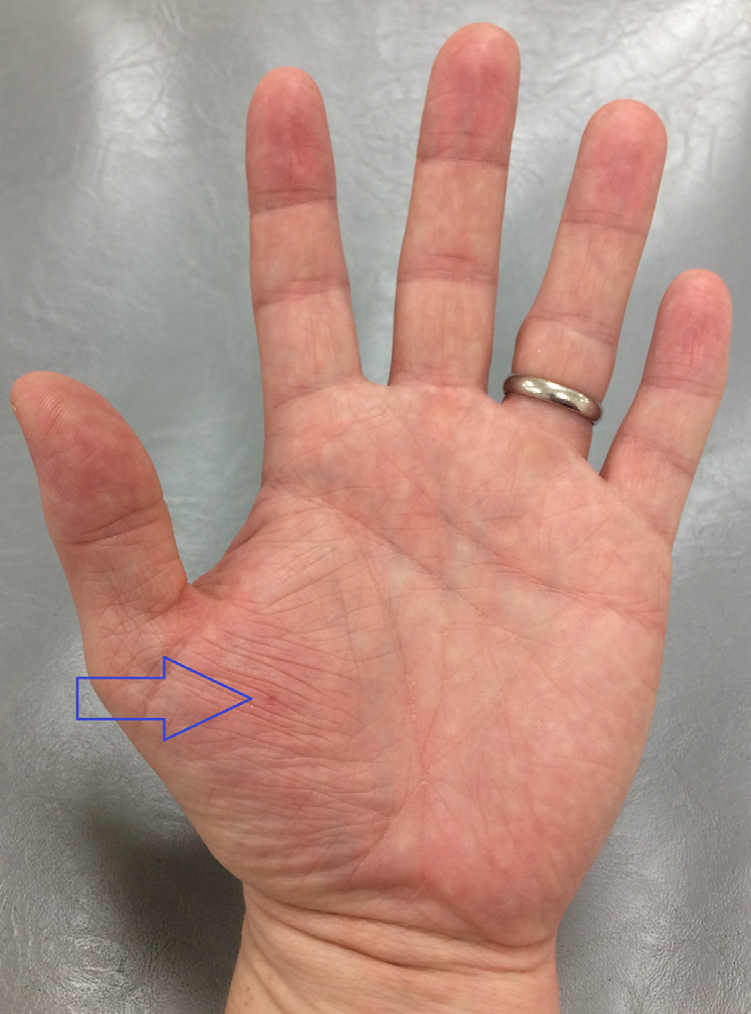
Site of needlestick injury (hollow blue arrow).

**Fig. 2:**
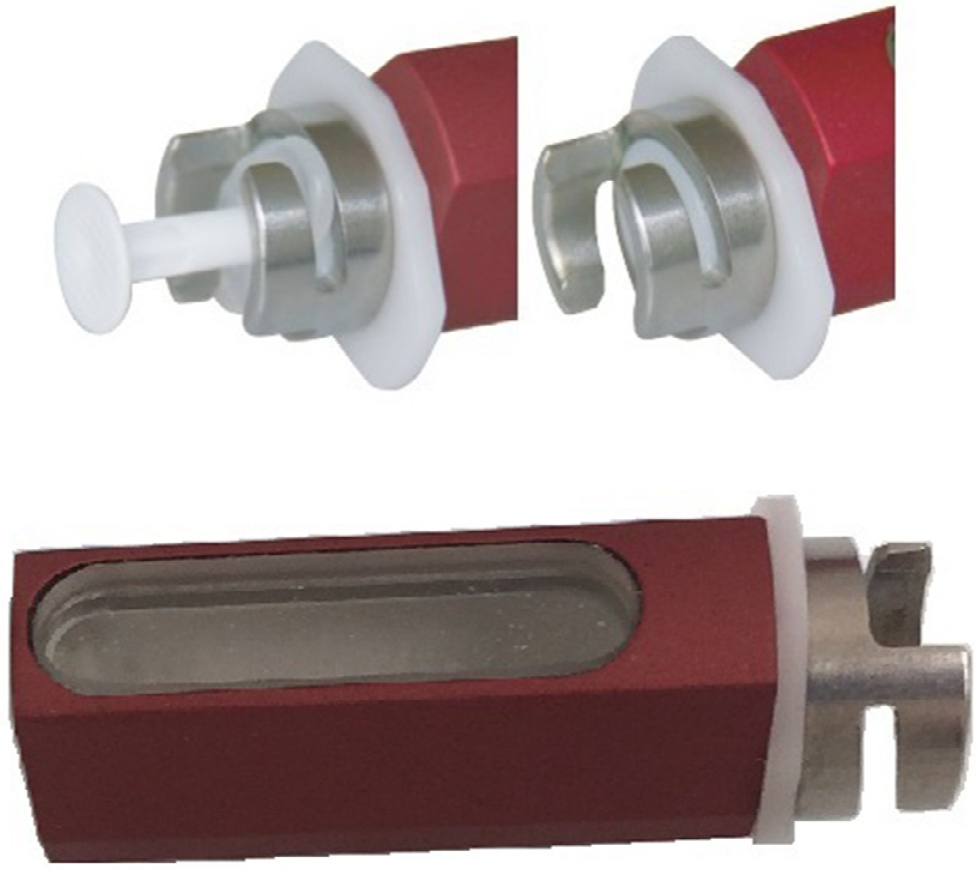
The Terumo Twist Lock Hoy lead syringe shield (HOY Scandinavian, Hadsund, Denmark) shows syringe twist-lock mechanism.

**Fig. 3:**
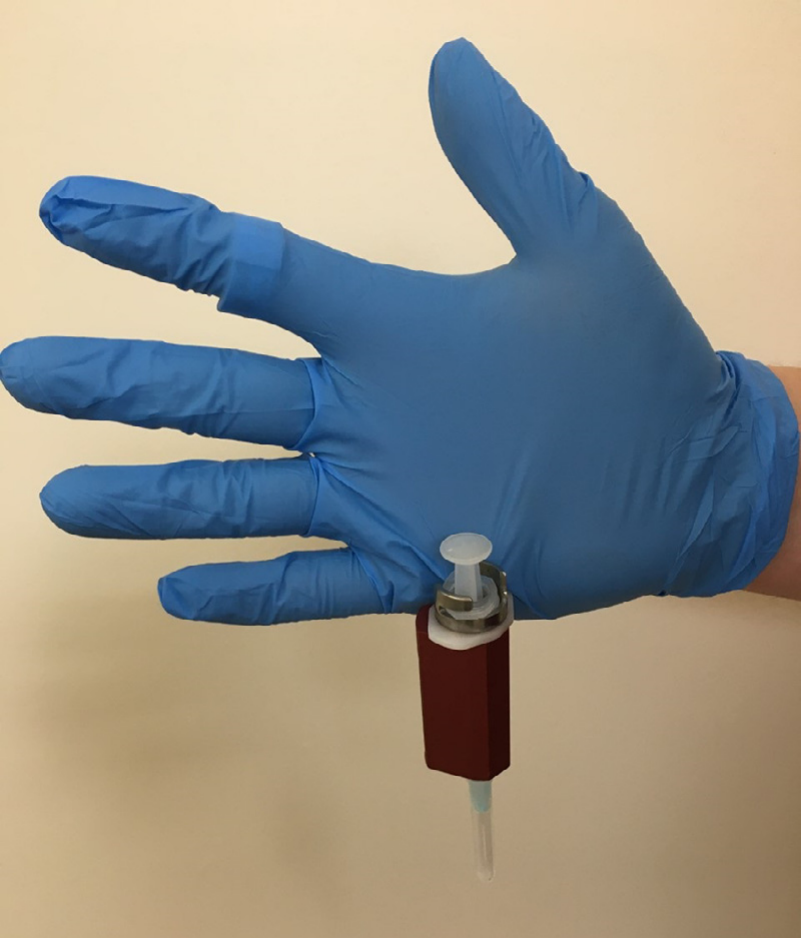
Recreation of glove entrapment within twist-lock mechanism.

During routine dispensing of ^99m^Tc-Tetrofosmin from a multi-dose vial, the operator (JE) incurred a needlestick injury to the thenar aspect of the left palm (Fig. 1). The target activity of 500 MBq was drawn up using a 2.5 mL syringe with a 23-gauge needle at a shielded dispensing station. Appropriate personal protective equipment had been used including tongs for handling of the vial, surgical gloves, and a 2.5 mL tungsten syringe shield. In addition, thermoluminescent dosimetry (TLD) finger rings and a whole-body badge were worn. The syringe shield (HOY Scandinavian, Hadsund, Denmark) utilizes a twist-lock mechanism whereby the proximal flange of the syringe is wedged into a fixed position (Fig. 2). Unfortunately, the surgical glove of the staff member was trapped within the mechanism during the re-capping procedure (Fig. 3). The unexpected pendulous motion of the apparatus subsequently led to a subcutaneous injury on the opposite hand which was promptly washed and encouraged to bleed.

**Fig. 4:**
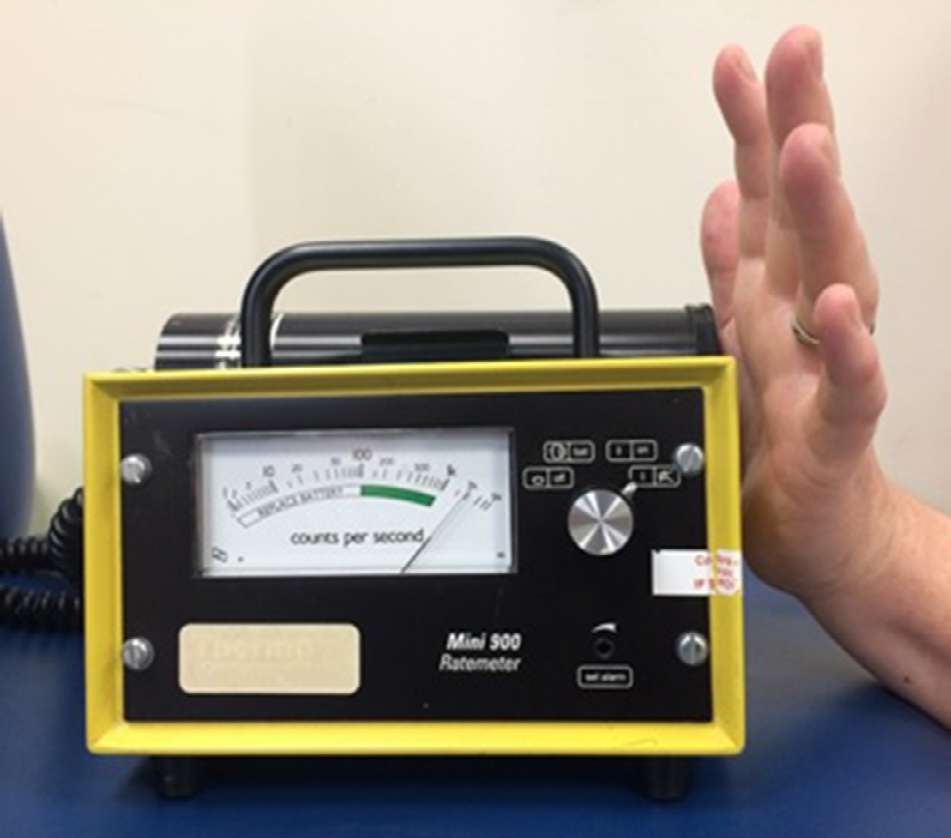
Contamination monitor (44A) registering 2000 counts per second.

**Fig. 5:**
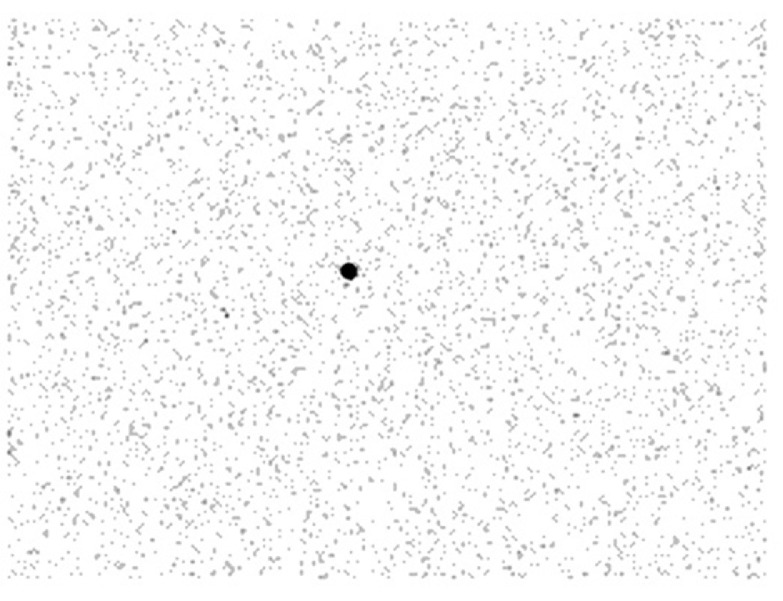
Scintigraphic imaging of needlestick injury site was taken 2 hours post injury.

Contamination monitoring indicated 2000 counts per second when in close proximity to the injury site (Fig. 4). Due to the novelty of opportunity, a 5-minute planar image of the palm was undertaken 2 hours post-injury on a General Electric Discovery 670 NM/CT using Low-Energy-High-Resolution collimators with suitable energy windowing for ^99m^Tc (140 keV ± 10%). Statistical analysis of the scintigraphic imaging (Fig. 5) demonstrated a ratio of 43:1 increase in radioactivity when compared to background counts, with total counts of the injury site exceeding 50,000 over the 5-minute acquisition. Deposited activity was estimated to be approximately 10 kBq in 0.2 µL, resulting in a negligible effective dose.

## Discussion

Recognition must be given to radiopharmaceutical NSI as a threat which is distinct from cross-infection risks associated with blood-borne pathogens. The dangers of ionizing radiation are well known, and localized doses have been associated with tissue damage and necrosis in severe cases [7,8]. In this case, no negative radiobiological or infective effects were observed. Although this incident incurred a low dose to the operator, the accident was avoidable, perhaps encouraging the use of single-dose syringes rather than multi-dose vials for multiple patient.

The importance of adequate hand protection within nuclear medicine has recently been raised with consideration for radiological and biological hazards [9], however, examples of surgical glove entrapment are lacking. A core principle used within radiation protection involves the use of shielding, distance, and reduced time to minimize exposure to ionizing radiation [10]. The drawing-up of radiopharmaceuticals necessitates direct handling of radioactivity and therefore an adequate syringe shield is essential along with a swift performance of radiopharmacy activities. Combined with finger TLD use, the operator is typically presented with bulky surgical gloves which ironically pose an entrapment risk with twist-lock syringe shields. Although complicit in the accident, the surgical glove may have reduced the transmission of radiopharmaceutical to the operator by acting as a barrier. Ironically, the practice of double-gloving in healthcare procedures has been shown to reduce needlestick fluid transmission further [11] but would be impractical for radiopharmaceutical dispensing.

Scintigraphic imaging demonstrated minimal biological clearance, with a focal point of activity suggestive of non-communication with lymphatic or blood circulation. Depending upon pushing force and accidental depression of the syringe plunger, the injury could have been deeper with a higher quantity of radiopharmaceutical administration. In theory, NSI to the dorsal aspect of the hand may have increased risks of vascular injury or systemic circulation of radiopharmaceutical due to the superficial nature of blood vessels. This case report has a noteworthy limitation of data collection which restricts the ability to perform multiple statistical calculations and thus reduces overall confidence in the quantification of radioactivity and subsequent dose to the operator. Nonetheless, the effective dose to the operator was deemed to be negligible.

Application of this experience upon clinical practice may be more relevant for α-particle emitting molecular radiotherapy nuclides such as ^223^Ra where consideration of radiobiological effects and operator dose would be far greater. In such instances, shielding of the needlestick injury site would be unrealistic, nor would attempts attempt to increase the distance from radio-sensitive organs. A pragmatic approach would include accurate dosimetry and clearance monitoring seen in radiopharmaceutical extravasation [12], with consideration for soft tissue excision in severe cases [8]. Suggestions for future practice include immediate disposal and replacement of needles involved in NSI, with any single-use equipment contaminated with staff bodily fluids to be discarded (including syringe contents). Furthermore, multi-use equipment such as the lead syringe shield should be diligently cleaned. A final recommendation is a careful and calm approach to radiopharmacy activities and the reassignment of staff with potentially detrimental conditions such as poor vision, hand tremors, or compromised hand-eye coordination.

## Conclusion

Radiopharmaceutical NSI during dispensing of radionuclides constitutes a risk that is distinct from blood-borne cross-infection threats during patient interaction. This case report provides a rare example of a radiopharmaceutical NSI with scintigraphic imaging and dose quantification. Whilst undoubtedly a freak accident, this study serves as a cautionary warning that care must be taken when drawing up radiopharmaceuticals for patient administration. The safe disposal of compromised equipment is advised, or effective cleaning thereof. Further caution may be advised with handling of α-particle emitting radionuclides which may impart greater radiobiological harm in subcutaneous injuries.

## Patient consent statement

This case report details the accidental needlestick injury of a member of staff (the primary author).

Full informed consent has been given for the publication of this case report.

## References

[1] Elseviers MM, Arias-Guillén M, Gorke A, Arens H-J. (2014). Sharps injuries amongst healthcare workers: Review of incidence, transmissions and costs. J Ren Care.

[2] Auta A, Adewuyi EO, Tor-Anyiin A, Edor JP, Kureh GT, Khanal V (2018). Global prevalence of percutaneous injuries among healthcare workers: a systematic review and meta-analysis. Int J Epidemiol.

[3] Deipolyi AR, Prabhakar AM, Naidu S, Oklu R. (2017). Needlestick injuries in interventional radiology are common and underreported. Radiology.

[4] UK Radiopharmacy Group (2013). https://cdn.ymaws.com/www.bnms.org.uk/resource/resmgr/guidelines/recapping_needles_ukrg_guida.pdf.

[5] Hung JC, Krause SJ, Schmit CL. (1999). Sensible approaches to avoid needle stick accidents in nuclear medicine. J Nucl Med Technol.

[6] Dewhirst CA, Hung JC. (2008). Comparison of the EZ-Cap recapper with the Mayo recapper for the prevention of needlesticks. J Nucl Med Technol.

[7] Vaiserman A, Koliada A, Zabuga O, Socol Y. (2018). Health impacts of low-dose ionizing radiation: current scientific debates and regulatory issues. Dose Response.

[8] van der Pol J, Vöö S, Bucerius J, Mottaghy FM. (2017). Consequences of radiopharmaceutical extravasation and therapeutic interventions: a systematic review. Eur J Nucl Med Mol Imaging.

[9] Ohnuki K, Yoshimoto M, Fujii H. (2021). Radiological protection and biological COVID-19 protection in the nuclear medicine department. Eur J Nucl Med Mol Imaging.

[10] Kim JH. (2018). Three principles for radiation safety: time, distance, and shielding. Korean J Pain.

[11] Din SU, Tidley MG. (2014). Needlestick fluid transmission through surgical gloves of the same thickness. Occup Med.

[12] Osborne D, Kiser JW, Knowland J, Townsend D, Fisher DR. (2021). Patient-specific extravasation dosimetry using uptake probe measurements. Health Phys.

